# The Role of Casein Phosphopeptide-Amorphous Calcium Phosphate (CPP-ACP) in White Spot Lesion Remineralization—A Systematic Review

**DOI:** 10.3390/jfb16080272

**Published:** 2025-07-25

**Authors:** Valentina Baccolini, Lígia Pereira da Silva, Liliana Teixeira, Raquel Teixeira de Sousa, Patrícia Manarte-Monteiro

**Affiliations:** 1 FP-I3ID, Faculty of Health Sciences, University Fernando Pessoa, 4200-150 Porto, Portugal; 2 RISE-Health, University Fernando Pessoa, 4200-150 Porto, Portugal

**Keywords:** amorphous calcium phosphate, casein phosphopeptide-amorphous calcium phosphate, bioceramic material, enamel, white spot lesions, remineralization

## Abstract

Amorphous calcium phosphate (ACP) is a well-established bioceramic material known to promote the remineralization of dental hard tissues. White spot lesions (WSLs) represent the initial stage of enamel demineralization and are frequently observed in patients with fixed orthodontic appliances or inadequate oral hygiene. Although recommendations for remineralizing agents include both the prevention of lesion progression and the stimulation of tissue remineralization, the clinical efficacy of ACP-based materials remains under debate. This systematic review, registered in the PROSPERO database (CRD42024540595), aims to evaluate the clinical efficacy of casein phosphopeptide-amorphous calcium phosphate (CPP-ACP)-based products in the remineralization of WSLs and to compare these outcomes with those achieved using non-bioceramic approaches. Inclusion criteria comprised randomized clinical trials, prospective cohort studies, and pilot studies conducted on human subjects with WSLs affecting permanent teeth. Studies involving artificial WSLs or non-cariogenic enamel lesions were excluded. The quality of the included studies was assessed using the Cochrane Risk of Bias 2 (RoB 2) tool. Fourteen articles met the inclusion criteria and were analyzed. The main findings indicate that CPP-ACP is clinically effective in promoting the remineralization of WSLs, although the results were inconsistent across studies. Comparisons with placebo and resin infiltration treatments revealed greater efficacy for CPP-ACP. The combination of CPP-ACP with fluoride appeared to further enhance the remineralizing effect on WSLs. Additional standardized clinical studies with longer follow-up periods are warranted to confirm these outcomes.

## 1. Introduction

White spot lesions (WSLs) are one of the first clinical signs of dental enamel demineralization, often observed in patients with fixed orthodontic appliances or with poor oral hygiene [1]. A systematic review and meta-analysis published in 2024, stated that the combined prevalence of WSLs among orthodontic patients was found to be 55.06%, whereas for those who did not require such appliances, the prevalence was 29.1% [2].

The main etiology of WSLs is linked to bacterial plaque activity and is considered as an initial stage of carious lesion development [3,4]. These lesions mainly appear on the vestibular surfaces of the lateral incisors, followed by the canines, premolars, and central incisors [5]. Clinically, WSLs are characterized by a whitish and opaque appearance, and may be found in various areas of the tooth such as on the fissures, fossae, and smooth surfaces [6]. Detection is typically achieved through simple visual inspection [7].

Diagnostic tools such as the International Caries Detection and Assessment System (ICDAS) help to classify the severity of carious lesions based on the dental hard tissues affected [8]. However, the clinical visualization of WSLs may pose some difficulties, and to aid in this aspect, more precise and reliable tools have been developed to be used in conjunction with visual assessments [9]. One such system is DIAGNOdent (Kavo; Biberach; Germany), which, through the application of laser-induced fluorescence, is capable of detecting caries with high sensitivity [9,10].

The treatment of WSLs with fluoride shows significant efficacy in preventing the progression of lesions, although it comes with some risks related to possible hypermineralization [3]. Casein phosphopeptide-amorphous calcium phosphate (CPP-ACP) has emerged as a promising alternative, as it stimulates remineralization by releasing calcium and phosphate ions on the tooth surface [11]. CPP-ACP is a biologically active material that functions through a technology in which casein binds to bacterial plaque, preventing bacteria from adhering to the surface and delaying biofilm formation. This product remineralizes the tooth surface via its role as a reservoir of calcium and phosphate, and when combined with fluoride ions, it enhances its precipitation on the dental hard surfaces [6,11,12].

Different forms of CPP-ACP-formulated toothpastes have been introduced to the market, like GC MI^®^, GC MI Plus^®^, or GC Tooth Mousse^®^ Plus (Recaldent™, GC Dental, Alsip, IL, USA) [13]. GC Tooth Mousse^®^ Plus is a commercially available product that contains a formula of ACP fluoride combined with CPP-ACP, which ultimately forms casein phosphopeptide-amorphous calcium phosphate fluoride (CPP-ACPF) [1]. The remineralization of WSLs through CPP-ACP is supported by several in vitro and clinical studies [6,14]. Furthermore, the association of CPP-ACP with fluoride has been shown to further enhance the remineralizing effects [12].

Other treatment options for WSLs include the use of fluoride-based-products or the application of infiltrating resins, such as the ICON^®^ (DMG, Hamburg, Germany) system, which utilizes low-viscosity resins to penetrate lesions and interrupt caries progression [15]. Although this technique offers aesthetic and mechanical advantages, evidence on its efficacy in improving remineralization remains limited [16].

Despite the various remineralizing strategies available—including fluoride varnishes and infiltrating resins—there is no clear consensus regarding the clinical superiority of CPP-ACP-based products [17]. This review aims to address the gap in the recent literature by focusing exclusively on the clinical efficacy of CPP-ACP-based products in treating naturally occurring WSLs and to compare these outcomes with those achieved using non-bioceramic approaches. By synthesizing the current evidence and identifying methodological gaps, this review seeks to clarify the potential role of CPP-ACP in managing early enamel lesions.

## 2. Materials and Methods

This systematic study was registered (CRD42024540595) in the International Prospective Register of Systematic Reviews (PROSPERO) and followed the Preferred Reporting Items for Systematic Review and Meta-Analysis (PRISMA) guidelines [18,19].

The research question, based on the PICO model, was as follows: Is there sufficient scientific evidence to support the efficacy and justify the clinical use of the CPP-ACP bioceramic materials for white spot lesions remineralization in erupted permanent dentition?

### 2.1. Search Strategy

A methodical search was conducted in three electronic databases (PubMed, Web of Science, and Google Scholar) covering the period from November 2023 to May 2024. The search strategy combined relevant keywords and Medical Subject Headings (MeSH) such as “molar”, “premolar”, “incisor”, “CPP-ACP”, “white spot lesions”, “remineralization”, and “follow-up studies”. Boolean operators (AND, OR) were applied according to the PICO framework to ensure comprehensive coverage of the research question. The full search strings for each database are presented in Table 1.

### 2.2. Studies Screening

All records retrieved were exported to EndNote v21 to remove duplicates. Two independent reviewers (V.B. and L.P.S.) screened the titles and abstracts to assess eligibility based on the predefined inclusion and exclusion criteria. The full texts of potentially relevant studies were then reviewed in detail. Any disagreements during the screening process were resolved through discussion and consensus, or by consulting a third reviewer (P.M.-M.), when necessary.

### 2.3. Inclusion Criteria, Exclusion Criteria, and Eligibility

The inclusion criteria were as follows: (1) study type (randomized controlled trials, prospective cohort studies, or pilot clinical studies); (2) human participants aged ≥11 years with WSLs on erupted permanent teeth; (3) studies evaluating CPP-ACP-based products alone or with fluoride; (4) comparators including a placebo, fluoride-based products, or infiltrating resins; (5) assessment of WSL remineralization using validated measures like visual inspection, photography, or DIAGNOdent; (6) minimum 4-week follow-up. Only English language studies published in the last 20 years were reviewed.

Studies that treated artificial WSLs, fluorosis, molar incisor hypomineralization (MIH), amelogenesis imperfecta, non-medical-dental materials, and studies without a follow-up period or absence of a control group in the study protocol were excluded.

Studies were collected and analyzed according to the following PICO strategy: *Population:* human teeth with white spot lesions; *Intervention:* application of bioceramic-based-CPP-ACP remineralizing products; *Comparison:* non-bioceramic products applied for remineralization purposes; *Outcomes:* surface remineralization of WSLs.

#### Justification for Criteria Selection

Inclusion and exclusion criteria were established to synthesize clinical data of high external validity. While in vitro studies offer valuable preliminary insights, they cannot reproduce the complexity of the oral cavity, including factors such as salivary flow, dietary influences, biofilm dynamics, and patient behavior. The exclusion of artificial lesions and in vitro models was therefore intended to enhance the applicability of the findings to everyday clinical practice, despite reducing the number of eligible studies.

### 2.4. Study Data

A bibliometric analysis was conducted, documenting the authors and the publication year. The examination methodology encompassed the objectives of each study, the materials and methods used, and the primary outcomes for both the experimental and control groups. Data extraction was carried out by two examiners (V.B. and L.P.S.), and a secondary examiner evaluated this phase (P.M.-M.).

### 2.5. Risk of Bias Assessment of Each Selected Study

The risk of bias for each included randomized controlled trial was assessed using the Cochrane Risk of Bias 2 (RoB 2) tool [20]. This tool evaluates bias across five domains: (1) bias arising from the randomization process, (2) bias due to deviations from intended interventions, (3) bias due to missing outcome data, (4) bias in measurement of the outcome, and (5) bias in selection of the reported result. Each domain is rated as having “low risk of bias”, “some concerns”, or “high risk of bias”, leading to an overall judgment for each study.

Two independent reviewers (V.B. and L.P.S.) conducted the assessments; a third reviewer evaluated the results (P.M.-M.). Any conflicts that occurred during this process were settled through discussion and consensus, or by seeking the input of a third reviewer (P.M.-M.), when needed. This approach allowed for a systematic and comprehensive evaluation of methodological rigor and provided insights into the reliability of the reported outcomes across the included studies.

## 3. Results

### 3.1. Studies Selection and Flow Diagram

**Figure 1:**
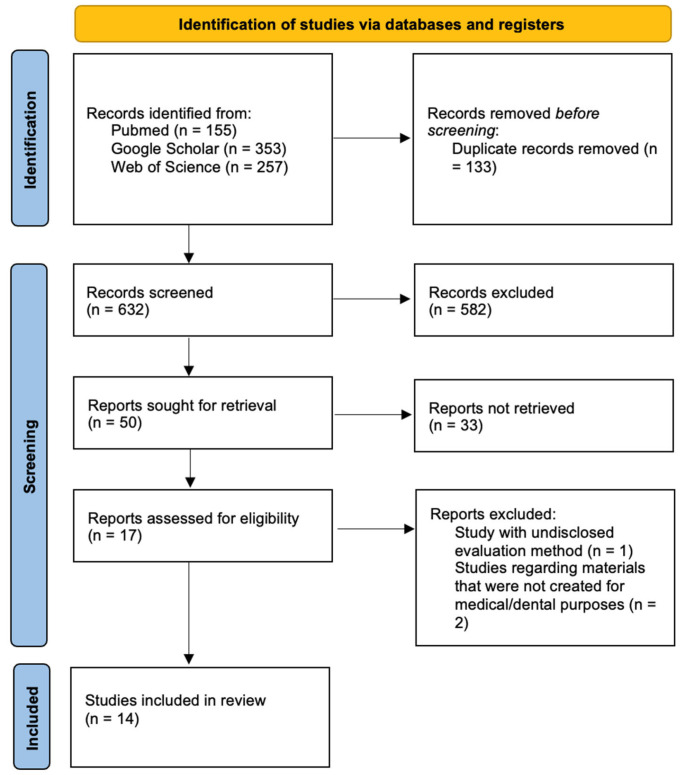
PRISMA 2020 flow diagram for new systematic reviews, which included searches of databases and registers only.

In total, 765 publications were screened through electronic databases searches (Figure 1). After removing duplicates, 632 articles were chosen for title evaluation, and 50 articles proceeded to abstract review and discussion.

Following the screening process, 17 publications were reviewed at the full-text level. Three of those studies were excluded due to failure to meet the inclusion criteria. Ultimately, 14 studies were deemed eligible and were included in this review.

### 3.2. Quality Assessment of the Included Studies

Most of the included studies demonstrated a low risk of bias in the domains of randomization and handling of missing data, while some concerns remained regarding lack of blinding and incomplete reporting of prespecified outcomes. Notably, four studies were rated as low risk across all domains, reinforcing the methodological robustness of part of the evidence base. A detailed evaluation of the methodological quality of the studies is shown in Table 2.

Overall, most trials demonstrated a low risk of bias in domains related to randomization and missing outcome data (Huang et al., 2013 [21], Krithikadatta et al. [9], Rechmann et al. [22], Wang et al., 2023 [23]). However, some studies presented “some concerns” in the overall assessment of risk of bias (Llena-Puy, 2013 [24], Yazicioğlu et al., 2017 [25], Baafif et al., 2020 [16], Mishra et al., 2023 [26], Mahmood et al., 2023 [27]), which may affect the reliability of their findings.

**Table 2:** Methodological quality assessment of the studies included in this review using the Cochrane Risk of Bias 2 (RoB 2) tool [20]. The assessment was performed regarding five main domains: 1—bias from randomization process; 2—bias due to deviations from intended interventions; 3—bias due to missing outcome data; 4—bias in measurement of the outcome; 5—bias in the selection of the reported result.

Study (Author, Year)	Risk of Bias Assessment Domain (RoB 2 Tool)					Overall Risk of Bias
	1	2	3	4	5	
Bailey et al., 2009 [28]	Some concerns	Low	Low	Some concerns	Low	**Low**
Robertson et al., 2011 [29]	Some concerns	Low	Low	Some concerns	Low	**Low**
Huang et al., 2013 [21]	Low	Low	Low	Some concerns	Low	**Low**
Llena-Puy, 2013 [24]	Some concerns	Low	Some concerns	Some concerns	Low	**Some concerns**
Krithikadatta et al., 2013 [9]	Low	Low	Low	Some concerns	Low	**Low**
Singh et al., 2016 [30]	Some concerns	Low	Low	Some concerns	Low	**Low**
Yazicioğlu et al., 2017 [25]	Some concerns	Low	Some concerns	Some concerns	Low	**Some concerns**
Karabekiroğlu et al., 2017 [31]	Some concerns	Low	Low	Some concerns	Low	**Low**
Beerens et al., 2018 [32]	Some concerns	Low	Low	Some concerns	Low	**Low**
Rechmann et al., 2018 [22]	Low	Low	Low	Some concerns	Low	**Low**
Baafif et al., 2020 [16]	Low	Some concerns	Some concerns	Some concerns	Low	**Some concerns**
Mishra et al., 2023 [26]	Some concerns	Low	Some concerns	Some concerns	Low	**Some concerns**
Mahmood et al., 2023 [27]	Low	Some concerns	Some concerns	Some concerns	Low	**Some concerns**
Wang et al., 2023 [23]	Low	Low	Low	Some concerns	Low	**Low**

### 3.3. Main Methodological Features of the Selected Trials

Of the fourteen studies included, only one was a pilot study (Krithikadatta et al. [9]); all others corresponded to randomized controlled trials. The participants’ ages ranged from 11 [29] to 56 years [23]. The study follow-up periods ranged from 4 weeks [25] to 3 years [31]. Thirteen studies (Huang et al. [21], Bailey et al. [28], Mahmood et al. [27], Krithikadatta et al. [9], Wang et al. [23], Karabekiroğlu et al. [31], Singh et al. [30], Beerens et al. [32], Robertson et al. [29], Rechmann et al. [22], Llena-Puy [24], Mishra et al. [26], Yazicioğlu et al. [25]) applied blind randomization for the experimental and control groups, while one study (Baafif et al. [16]) used the split-mouth design. Most WSLs occurred in the vestibular tooth surfaces, except in the studies of Krithikadatta et al. [9] and Yazicioğlu et al., 2017 [25], where the occlusal surfaces were analyzed. Seven studies [9,23,26,27,28,30,31] tested CPP-ACP, and nine studies [9,16,21,22,24,25,26,29,32] tested CPP-ACPF.

Two studies [9,26] combined both based-substances as experimental groups. Four study [23,28,29,32] designs used a placebo as the control group. In all studies, CPP-ACP-based-products were provided by the research team to participants in a toothpaste formula.

Six studies [9,16,25,26,30,31] evaluated the surface remineralization of WSLs using the DIAGNOdent Pen (KaVo Dental GmbH, Warthausen, Germany), which measures remineralization via laser-induced fluorescence. Five studies [21,23,24,29,32] used digital photography to assess the surface lesions; four studies [22,25,27,28] evaluated the dental surfaces exclusively via visual examination.

Table 3 provides a summary of the selected studies, along with their main details.

**Table 3:** Summary of core characteristics of the 14 included clinical studies evaluating CPP-ACP-based treatments. The table includes sample size, tooth surface location of enamel lesion, evaluation method, and follow-up duration.

Study (Author, Year)	Sample Size (N)	WSLs Surface Location	Evaluation Method	Follow-Up Period
Bailey et al., 2009 [28]	45	NS	ICDAS	12 weeks
Robertson et al., 2011 [29]	50	NS	Photo (NS) + ICDAS	3 months
Beerens et al., 2018 [32]	51	Vest.	Photo (NS) + ICDAS	12 months
Wang et al., 2023 [23]	79	Vest.	Photo (Image J, version 1.52e)	12 months
Krithikadatta et al., 2013 [9]	45	Occl.	DIAGNOdent	30 days
Singh et al., 2016 [30]	45	NS	DIAGNOdent + ICDAS	5 months
Yazicioğlu et al., 2017 [25]	30	Occl.	DIAGNOdent + ICDAS	4 weeks
Karabekiroğlu et al., 2017 [31]	41	Vest.	DIAGNOdent	3 years
Rechmann et al., 2018 [22]	40	NS	ICDAS	12 months
Llena-Puy, 2013 [24]	135	Vest.	Photo (Image J, version information not given)	8 weeks
Huang et al., 2013 [21]	115	Vest.	Photo (NS)	8 weeks
Mahmood et al., 2023 [27]	126	NS	ICDAS	56 days
Mishra et al., 2023 [26]	75	Vest.	DIAGNOdent	12 months
Baafif et al., 2020 [16]	28	Vest.	DIAGNOdent	12 months

N—number of participants; Vest.—vestibular surface of the tooth; Occl.—occlusal surface of the tooth; WSLs—White spot lesions; NS—not specified.

### 3.4. Clinical Efficacy Outcomes of CPP-ACP and Other Non-Bioceramic Aproaches

#### 3.4.1. Efficacy of CPP-ACP Compared to That of Placebo Treatments

The studies of Bailey et al. [28], Wang et al. [23], Beerens et al. [32], and Robertson et al. [29] analyzed the effect of CPP-ACP toothpaste compared to that of placebo treatments. When comparing CPP-ACP to the placebo, the results were mixed: some studies reported clear benefits, while others found no significant difference, often attributing remineralization to saliva alone.

Bailey et al. [28] and Robertson et al. [29] reported satisfactory results for WSLs remineralization. Bailey et al. [28] stated a 72% regression of lesions (versus 58.7% for placebo); Robertson et al. [29] identified a 44.8% regression of lesions (versus 43.1% for placebo). In contrast, in the work of Wang et al. [23] and Beerens et al. [32], no significant statistical differences were found between the results for the experimental and control groups. The methodology, evaluation methods, and clinical outcome comparisons are reported in Table 4.

**Table 4:** Summary of all included clinical studies comparing CPP-ACP-based treatments with placebo, fluoride, or resin infiltrates for the management of WSLs. The table includes study characteristics, population types, evaluation methods, and clinical interpretations to support a more analytical understanding of the comparative efficacy.

Study (Author, Year)	Population	Comparison	CPP-ACP Formulation	Clinical Interpretation
Bailey et al., 2009 [28]	Orthodontic patients	Placebo	GC MI^®^	CPP-ACP reduced lesions by 72%
Robertson et al., 2011 [29]	Adolescents	Placebo	GC MI Plus^®^	CPP-ACP slightly more effective than placebo
Beerens et al., 2018 [32]	Post-orthodontic adults	Placebo	GC MI Plus^®^	No significant difference; effect possibly due to saliva
Wang et al., 2023 [23]	Adolescents/young adults	Placebo	GC Tooth Mousse^®^ Plus	No significant difference
Krithikadatta et al., 2013 [9]	Orthodontic adolescents	Fluoride	GC MI^®^/GC MI Plus^®^	CPP-ACPF showed superior remineralization
Singh et al., 2016 [30]	Orthodontic patients	Fluoride	GC Tooth Mousse^®^ Plus	No significant difference
Yazicioğlu et al., 2017 [25]	Orthodontic adolescents	Fluoride	GC MI Plus^®^	CPP-ACP slightly more effective than fluoride
Karabekiroğlu et al., 2017 [31]	Orthodontic patients	Fluoride	GC Tooth Mousse^®^ Plus	Comparable to fluoride; long-term stability noted
Rechmann et al., 2018 [22]	Orthodontic patients	Fluoride	GC MI Plus^®^	No added benefit
Llena-Puy, 2013 [24]	Adults	Fluoride	GC MI Plus^®^	No significant difference
Huang et al., 2013 [21]	Adolescents/young adults	Fluoride	GC MI Plus^®^	No significant difference
Mahmood et al., 2023 [27]	Orthodontic patients	Fluoride	GC MI^®^	No significant difference
Mishra et al., 2023 [26]	Orthodontic patients	Fluoride	GC MI^®^/GC MI Plus^®^	CPP-ACPF showed superior remineralization
Bailey et al., 2009 [28]	Orthodontic patients	Resin	GC MI Plus^®^	CPP-ACP more effective than resin

N—number of participants; NS—not specified; CPP-ACP—casein phosphopeptide-amorphous calcium phosphate.

#### 3.4.2. Efficacy of CPP-ACP Compared to Fluoride Application Approaches

Nine studies [9,21,22,24,25,26,27,30,31] compared CPP-ACP toothpaste with fluoride toothpaste applications in treating WSLs. The comparisons with fluoride treatments generally revealed comparable efficacy, although a few studies noted enhanced outcomes when CPP-ACP was combined with fluoride. Tooth surface remineralization and enamel spot improvements were detected by DIAGNOdent Pen (Kavo Dental GmbH) but with no clear superiority of CPP-ACP-based materials over fluoridated products, as comparisons finding reported in Table 4.

#### 3.4.3. Efficacy of CPP-ACP Compared to Infiltrating Resins Applications

Only one study, that of Baafif et al. [16], compared the CPP-ACP toothpaste (GC MI Plus^®^, Recaldent™, GC Dental, Alsip, IL, USA) with infiltrating resins (ICON^®^, DMG, Hamburg, Germany). The authors observed better results after the use of CPP-ACP toothpaste (*p* < 0.001) and concluded that its effect extended over a longer period. Comparison and clinical outcome are reported in Table 4.

## 4. Discussion

The rising incidence of dental caries and erosion has driven research in the dentistry field to identify more advanced preventive and therapeutic methods. Although traditional agents like fluoride have played a crucial role in promoting tooth remineralization, limitations in delivering bioavailable ions have been reported. Innovative strategies such as CPP-ACP have demonstrated potential in stabilizing these ions and improving the remineralization process [33].

The development of new products to prevent the development of early carious lesions has led to interest in substances capable of releasing active components during the dental hard tissues demineralization and remineralization processes [34]. CPP-ACP, a milk casein-derived compound, has shown efficacy in the remineralization of early carious lesions, such as WSLs, with good results regarding enamel demineralization prevention. Evaluated in experimental models such us animal, in vitro, in situ, and clinical studies, this bioactive material demonstrates potential as a remineralizer of tooth enamel [35].

This systematic review evaluated the efficacy of CPP-ACP-based-products in the remineralization of WSLs when compared to that of conventional approaches, i.e., fluoride-based-products and the use of infiltrating resin. Additionally, CPP-ACP’s efficacy was also reported when compared to that of placebo treatments. The 14 studies included in this review showed that CPP-ACP is indeed effective for WSLs remineralization, with varying results when compared to those for fluoride products, infiltrating resins, and placebo applications.

### 4.1. CPP-ACP Compared to Placebo Treatments

Several studies assessed the efficacy of CPP-ACP in comparison to placebo treatments, but these investigations revealed inconsistent results. Bailey et al. [28] observed that WSLs tended to spontaneously regress within 12 weeks with CPP-ACP use, but not with placebo. Wang et al. [23] found significant improvements in enamel surface remineralization in both groups (CPP-ACP and placebo), with no statistical differences between them. Beerens et al. [32] reported similar outcomes, attributing the WSLs remineralization to the action of the participant’s saliva. In contrast, Robertson et al. [29] and Thierens et al. [36] showed that the efficacy of CPP-ACP-based products was superior to those of the placebo. Oliveira et al. [37] showed that CPP-ACPF was more effective than CPP-ACP alone.

### 4.2. CPP-ACP Compared to Fluoride Treatments

When compared to fluoridated products, CPP-ACP also revealed inconsistent findings. While Yazicioğlu et al. [25] demonstrated a significant remineralization effect of CPP-ACP-based products, the results of LIena-Puy [24], Beerens et al. [12], Huang et al. [21], and Rechmann et al. [22] were not as satisfactory, suggesting that CPP-ACP was not more effective than fluoride in the tooth remineralization process. The findings of Mahmood et al. [27] revealed that CPP-ACP showed good results when compared to those for fluoride but without overall superiority. The literature also indicates that CPP-ACP treatments show additional benefits, such as better safety and lower risk of biotoxicity [9,21,22,24,25,26,27,30,31]; however, it may cause rare allergic reactions [6].

Some studies suggested that the combination of CPP-ACP and fluoride may offer greater benefits [1,38], revealing a synergistic effect. Singh et al. [30] reported the benefits of combined treatment with CPP-ACP and fluoride varnish. Karabekiroğlu et al. [31] found that CPP-ACP and low-concentration fluoridated toothpaste were equally effective in preventing WSLs in the long-term evaluations. Mishra et al. [26] suggested that, in orthodontic patients with high caries risk, the combined use of CPP-ACP and fluoride may offer significant advantages in preventing enamel demineralization. Rajendran et al. [39] reported that the combination of the two products improved fluoride absorption and tooth remineralization. Ma et al. [6] emphasized the need for further studies to evaluate the efficacy of CPP-ACP without the influence of fluoride-products applications, emphasizing the safety of CPP-ACP compared to the risks associated with fluoride, such as fluorosis.

### 4.3. CPP-ACP Compared to Infiltrating Resins

CPP-ACP findings were also compared to results obtained after applying infiltrating resins, such as the ICON^®^ (DMG, Hamburg, Germany) system. Although both treatments led to improvements in enamel surface hardness, CPP-ACP demonstrated a higher effect, with improved surface remineralization when used for prolonged periods of time. Baafif et al. [16] observed that, within a 12 month follow-up period, CPP-ACP was more effective than the ICON^®^ infiltrating resin. However, Pintanon et al. [40] suggested that infiltrating resins may be more effective in improving primarily aesthetics. Aref and Alrasheed’s research suggested that using CPP-ACP products, followed by adhesive resin infiltration, may effectively address WSLs by enhancing surface microhardness, improving aesthetics, and creating a smoother surface [41].

### 4.4. Clinical Implications and Patient-Centered Relevance

WSLs are a common concern in orthodontic patients due to plaque accumulation around brackets and difficulty maintaining oral hygiene [1]. CPP-ACP-based products offer a non-invasive option for managing and preventing these lesions. Their ability to deliver bioavailable calcium and phosphate directly to demineralized enamel makes them especially suitable for long-term use during orthodontic treatment [42]. Moreover, their favorable safety profile and ease of application support their use in young patients or individuals with limited compliance [43].

In summary, CPP-ACP can be a valid alternative to fluoride, but the combination of the two substances appears to be more effective, especially in orthodontic patients and those with high caries risk. Short-term studies (4 to 12 weeks) have shown that CPP-ACP may be effective in early carious lesions remineralization [9,25,28,29]. Nonetheless, long-term results, especially after 12 months, suggested that the efficacy of CPP-ACP may be lower than that observed in short-term follow-ups [22,32]. This review suggests that although CPP-ACP has shown good performance in short- and medium-term treatments, more long-term studies are needed to assess its continued efficacy and sustainability [44].

CPP-ACP-based products are a valid and effective option in the treatment of early carious lesions, offering an alternative to traditional fluoride formula applications The combination of CPP-ACP with other agents for tooth remineralization also deserves further investigation [6]. Combining it with fluoride and other treatments may also be promising, but more research is needed to determine the best clinical protocols and their long-term performance [30].

Compared to previous systematic reviews, this study includes the most recent clinical evidence and applies a more rigorous risk of bias assessment (RoB 2). In contrast to earlier reviews that often included in vitro or non-randomized studies, our analysis focuses exclusively on human clinical trials with well-defined diagnostic methods and follow-up durations, thereby improving the clinical relevance of the findings. This qualitative systematic review highlighted the need for additional randomized controlled trials (RCTs) with standardized protocols to evaluate the remineralization efficacy of CPP-ACP for the treatment of WSLs. Future studies should adopt uniform assessment methods, extended follow-up periods, and standardized diagnostic criteria to improve the reliability and comparability of the results. Incorporating variables such as ethnicity and socioeconomic status will enhance the generalizability of the findings. Moreover, exploring combinations of CPP-ACP with other agents, such as amorphous calcium phosphate, and assessing patient acceptance are important avenues for future research.

This review did not perform a meta-analysis due to considerable heterogeneity across studies, including differences in study designs, diagnostic methods, outcome measurements, and follow-up periods. Hence, a key limitation among the included studies was the heterogeneity in WSLs detection and evaluation methods, ranging from visual inspection to the use of digital and fluorescence-based tools (e.g., DIAGNOdent). This variability, along with inconsistent follow-up durations (4 weeks to 3 years), complicated direct comparisons and weakened the strength of the pooled conclusions. Standardized assessment protocols are therefore essential for future investigations. Distinct from previous reviews, this study focuses exclusively on clinical trials involving naturally occurring WSLs in permanent human dentition and systematically compared CPP-ACP with fluoride, resin infiltrates and placebo/controls. The review also critically addressed the impact of diagnostic heterogeneity on outcome interpretation.

Overall, CPP-ACP appeared to be an effective agent for the remineralization and prevention of early carious lesions and WSLs, with efficacy comparable to that of conventional fluoride treatments. Standardization in future research will be crucial to further clarify its clinical benefits and optimize its integration into dental care.

## 5. Conclusions

CPP-ACP emerges as a clinically relevant option for the remineralization of WSLs, particularly in orthodontic patients and individuals at high caries risk. Although the evidence remains somewhat inconsistent compared to that for fluoride-based products and infiltrating resin, CPP-ACP demonstrates notable potential as a remineralizing agent, especially in combination with fluoride. To enhance clinical decision making, future randomized controlled trials should adopt standardized outcome measures and extended follow-up durations and should also include diverse patient populations. A clearer understanding of the contexts in which CPP-ACP is most effective will support its integration into personalized caries management strategies.

## Figures and Tables

**Table 1:** Search strategy used in each electronic database.

PubMed		
(Molar [Title/Abstract]) OR (Premolar [Title/Abstract]) OR (Posterior teeth [Title/Abstract]) OR (Incisor [Title/Abstract])	(Casein amorphous calcium phosphate [Title/Abstract]) OR (CPP-ACP [Title/Abstract]) AND (Dental white spots [Title/Abstract]) OR (White spot lesions [Title/ Abstract]) OR (Dental caries [Title/Abstract])	(Remineralization [Title/ Abstract]) OR (Tooth remineralization [Title/Abstract]) AND (follow-up studies [Title/Abstract])
**Web of Science**		
topic: Molar* OR Bicuspid* OR Premolar* OR Posterior teeth* OR Incisor teeth*	topic: Casein amorphous calcium phosphate* OR CPP-ACP* AND Dental white spot* OR White spot lesion* OR Dental caries*	topic: Remineralization* OR Tooth remineralization* AND Follow-up studies*
**Google Scholar**		
In title: (Molar OR Premolar OR Posterior teeth OR Incisor teeth)	In title: (Casein amorphous calcium phosphate OR CPP-ACP AND Dental white spots OR White spot lesions OR Dental caries)	In title: (Remineralization OR Tooth remineralization AND Follow-up studies)

## Data Availability

The data presented in this study are available on request from the corresponding author as they consist of compiled data extracted from published articles and are not publicly archived.

## Abbreviations

The following abbreviations are used in this manuscript:

ACP	Amorphous calcium phosphate
CPP-ACP	Casein phosphopeptide-amorphous calcium phosphate
CPP-ACPF	Casein phosphopeptide-amorphous calcium phosphate fluoride
WSL	White spot lesion
